# The Clinical Features of Graves' Orbitopathy with Elevated Intraocular Pressure

**DOI:** 10.1155/2021/9879503

**Published:** 2021-01-25

**Authors:** Shu-Xian Fan, Peng Zeng, Zi-Jing Li, Jing Wang, Jia-Qi Liang, Yun-Ru Liao, Yu-Xin Hu, Ming-Tong Xu, Mei Wang

**Affiliations:** ^1^Department of Ophthalmology, Sun Yat-sen Memorial Hospital, Sun Yat-sen University, Guangzhou 510120, China; ^2^Department of Endocrinology, Sun Yat-sen Memorial Hospital, Sun Yat-sen University, Guangzhou 510120, China

## Abstract

**Background:**

To investigate the clinical characteristics of Graves' orbitopathy (GO) with elevated intraocular pressure (IOP) using the European Group of Graves' Orbitopathy (EUGOGO) system.

**Methods:**

In this retrospective study, the clinical data of GO patients with elevated IOP (≥21 mmHg) were collected in Sun Yat-sen Memorial Hospital from January 2010 to June 2016. The demographic characteristics, clinical history of thyroid disease and GO, and ocular examination data were evaluated, and the activity and severity of GO were classified.

**Results:**

Data were collected from 58 eyes of 39 patients. The durations of thyroid disease and GO were 15.9 ± 18.9 months and 7.5 ± 6.2 months, respectively. The average IOP was 24.8 ± 5.3 mmHg (range: 21–55 mmHg). No significant difference in IOP was observed between active and inactive eyes. Eight eyes (13.8%), 29 eyes (50.0%), and 21 eyes (36.2%) were graded as mild, moderate-severe, and sight-threatening disease, respectively, according to the EUGOGO classification. The IOP was not significantly different among the three EUGOGO grades. No glaucomatous optic nerve damage or visual field defects were found.

**Conclusion:**

Increased IOP was evident for every grade of GO severity and activity of the EUGOGO system. IOP, glaucomatous optic nerve damage, and visual fields must be evaluated regularly during follow-up evaluations, regardless of the degree of activity and severity of GO.

## 1. Background

Graves' orbitopathy (GO), also known as thyroid-related ophthalmopathy, is an autoimmune disease characterized by enlarged extraocular muscles and increased retrobulbar fat. The pathogenesis of GO is associated with lymphocyte infiltration and glycosaminoglycan deposits in the extraocular muscles, connective tissue, and orbital fat [1], which may cause clinical ophthalmopathy, including proptosis, diplopia, periorbital edema, corneal breakdown, and compressive optic neuropathy [2]. Elevated intraocular pressure (IOP) in GO was first reported by Wessely in 1918 [3], and since then, many studies have been conducted on the incidence and treatment of increased IOP [4–7]. Although elevated IOP directly associated with GO is a less common problem, it may result in damage to the optic nerve [8].

No physical signs or symptoms, only signs, soft-tissue involvement, proptosis, extraocular muscle signs, corneal involvement, and sight loss (NOSPECS) and the European Group of Graves' Orbitopathy (EUGOGO) classifications are popular grading systems for assessing the clinical severity of GO. However, the clinical characteristics of the elevated IOP have mainly been analyzed using the NOSPECS classification of GO, and few reports describing increased IOP in GO using the EUGOGO system are currently available. In this study, we analyzed elevated IOP in GO using the EUGOGO system and determined its relationship to data in the NOSPECS system.

### 1.1. Patients and Methods

The study was conducted in Sun Yat-sen Memorial Hospital, Sun Yat-sen University, Guangzhou, China, from January 2010 to June 2016. This study was conducted in accordance with the tenets of the Declaration of Helsinki, and the protocol of this study was approved by the Sun Yat-sen University Sun Yat-sen Memorial Hospital Ethical Committee in China. GO was diagnosed in the Department of Endocrinology and the Department of Ophthalmology based on clinical and laboratory findings [9]. The clinical data of 39 GO patients with elevated IOP (≥21 mmHg) and complete examinations were collected. Patients using any IOP-lowering agents or ocular drops that influence the ocular surface and those with a history of prior ophthalmic surgery, orbital decompression, or other immune disease were excluded.

The collected data included age, sex, signs, symptoms, duration of GO, clinical activity, and severity classification of GO according to the EUGOGO system, history and duration of thyroid disease, history of smoking, and presence of other systemic and eye diseases. Ocular examinations included best-corrected visual acuity, slit-lamp examination of the anterior segment, and fundus examination with *a* +90 lens. Proptosis was measured with the Hertel exophthalmometer. IOP measurements were performed in the primary position using a noncontact tonometer (NCT) (Canon TX-20, YZB/JAP3501-2012, Tokyo, Japan) or the Schiotz tonometer in cases of strabismus; each eye was measured 3 times and took the average in the morning time. Standard automated visual fields were obtained using the Humphrey automated visual field analyzer (program 30–2).

Glaucomatous changes in the optic nerve were defined as a cup disc ratio greater than 0.6, vertical cup asymmetry greater than 0.2, neuroretinal rim loss or notching with or without disc hemorrhages, and nerve fiber layer defects. According to the probability plot, visual fields were considered abnormal if 2 or more adjacent points were depressed ≥5 dB or if 1 point was depressed ≥10 dB compared to the surrounding points in any region of the central 30 degrees of the visual field, with a mean deviation greater than 2 dB, pattern standard deviation outside the 95% confidence interval, and a glaucoma hemifield test result outside the normal range.

Two grading systems were used to assess the severity of GO: the NOSPECS classification, which was described by Werner [10], and the EUGOGO classification [11], which includes the categories of mild, moderate-severe, and sight-threatening disease. The mild disease was defined as minor lid retraction (<2 mm), mild soft-tissue involvement, exophthalmos <3 mm above normal value for race and gender, no or intermittent diplopia, and corneal exposure that is responsive to lubricants. Moderate-severe GO was defined as lid retraction ≥2 mm, moderate or severe soft-tissue involvement or exophthalmos ≥3 mm above normal value for race and gender, and variable or constant diplopia. Sight-threatening GO was defined as dysthyroid optic neuropathy and/or severe corneal exposure (large epithelial and/or stromal defects) or corneal breakdown (descemetocele or frank perforation).

The activity of GO is measured by the clinical activity score (CAS) [11], which includes spontaneous retrobulbar pain, pain on attempted upward or downward gaze, redness of the eyelids, redness of the conjunctiva, swelling of the caruncle or plica, swelling of the eyelids, and swelling of the conjunctiva (chemosis). A CAS ≥3/7 is indicative of positive activity.

### 1.2. Statistical Analysis

Statistical analyses were performed using SPSS (Statistical Package for Social Sciences; SPSS Inc. IBM, Armonk, NY) version 22.0. Data are expressed as the means ± standard deviation. The significance of differences in the mean IOP according to gender, eye, and disease activity, and the mean proptosis value of the eye was assessed by *t*-tests (independent samples *t*-test or *t*' test). The Kruskal–Wallis test was used to evaluate significant differences in the mean IOP according to the EUGOGO classification and the proptosis classification in the NOSPECS system. Differences were considered statistically significant at *P* ≤ 0.05.

## 2. Results

### 2.1. Demographic and Other Characteristics

A total of 58 eyes of 39 GO patients were included in the analyses. The demographic and clinical patient characteristics, including the affected eye, gender, age, course of thyroid disease and GO, and smoking habits, are summarized in Table 1. Male patients (21 patients, 53.8%) were more than female patients (18 patents, 46.2%). The mean age was 49.8 ± 12.5 years, and male (49.5 ± 12.5 years) and female (50.2 ± 10.4 years) patients' mean age had no significant difference (*P*=0.109). The most common primary thyroid disease was hyperthyroidism (36 patients, 92.3%). The average durations of thyroid disease and GO were 15.9 ± 18.9 months and 7.5 ± 6.2 months, respectively. All patients with smoking habits were male. Most patients (28 patients, 71.8%) used antihyperthyroidism medication, and others had undergone treatments, including radioiodine therapy (7 patients) and thyroidectomy (4 patients). However, many patients (34 patients, 87.2%) still had hyperthyroidism, with only 17.9% of the patients exhibiting normal thyroid function at the time of ocular examination. No patients had received systemic corticosteroid treatment or orbital radiotherapy before the ocular examination.

### 2.2. Ocular Presentation

**Figure 1:**
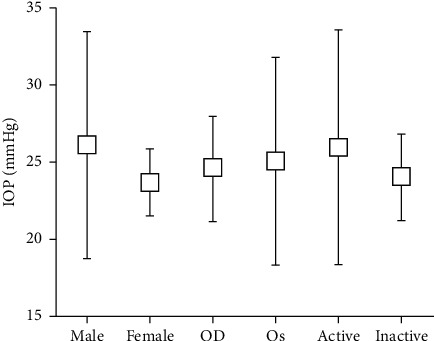
Box and whisker plot showing the distribution of intraocular pressure (IOP) for different genders, eyes, and disease activity grades. No significant difference in IOP was found between genders, eyes, and disease activity grades.

The mean IOP was 24.8 ± 5.3 mmHg (range: 21–55 mmHg). No significant difference in IOP was observed between genders or eyes (*P*=0.109, *P*=0.739; Figure 1). The mean IOP of smokers (27.38 ± 8.76 mmHg) was higher than that of nonsmokers (23.73 ± 2.45 mmHg, *P* < 0.00). Twenty-four eyes (41.1%) were active, with a CAS ≥3, and the remaining eyes were inactive, with a CAS<3. The mean IOP in the active eyes was 25.98 ± 7.56 mmHg, which was not significantly different from that in the inactive eyes (24.02 ± 2.79 mmHg, *P*=0.237) (Figure 1).

One patient was found to have disc swelling, but no glaucomatous optic nerve damage or visual field defects, such as arcuate scotoma, were found in any patients. All cup disc ratios in eyes with increased IOP were less than 0.6.

**Figure 2:**
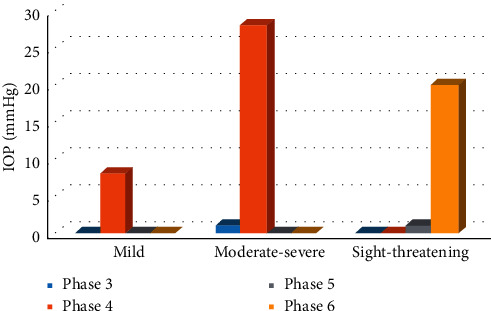
3D column chart comparing the distribution of high IOP in the NOSPECS and EUGOGO systems.

The distribution of eyes with elevated IOP was different according to the EUGOGO and NOSPECS systems. In the EUGOGO classification, eyes with elevated IOP were distributed among all scopes of severities, including grades of mild (8 eyes, 13.8%), moderate-severe (29 eyes, 50.0%), and sight-threatening (21 eyes, 36.2%). However, in the NOSPECS system, the distribution was mainly concentrated in phase 4 (36 eyes, 27 patients, 64.1%) and phase 6 (20 eyes, 13 patients, 34.5%). Most of the eyes in the mild or moderate-severe GO categories according to the EUGOGO system were classified as phase 4 and phase 6 according to the NOSPECS system (Figure 2). In addition, the IOP levels were not significantly different among various severities of the EUGOGO or NOSPECS system (Table 2).

The mean proptosis value in all eyes with increased IOP was 19.8 ± 3.2 mm (range: 14–28 mm), and the mean values were 19.9 ± 3.1 mm (range: 15–28 mm) in right eyes and 19.9 ± 3.3 mm (range: 14–28 mm) in left eyes. The exophthalmos degree was not significantly different between the right and left eyes. According to the classification of proptosis in the NOSPECS system [12], the IOP gradually increased with increased proptosis, although no statistically significant association was found between these factors (Table 3).

## 3. Discussion

This study aimed to explore the clinical features of elevated IOP in GO according to the EUGOGO system. The study revealed that elevated IOP was present in eyes with each severity degree of the EUGOGO system, from mild to sight-threatening, but it was more common in the higher stages of the NOSPECS system, including phases 4 and 6. Elevated IOP was present in both active and inactive eyes, and the mean IOP in the inactive eyes was comparable to that in the active eyes.

Similar to other reports [13, 14], the average age of all patients was 49.8 ± 12.5 years, and the average IOP was 24.8 ± 5.3 mmHg in this study. Cockerham et al. [14, 15] reported that the IOP in GO was greater than 22 mmHg but less than 30 mmHg. However, the present study identified 6 patients with IOP levels greater than 30 mmHg, and the highest IOP recorded was 55 mmHg, as measured with an NCT. This discrepancy may be due to differences in sample sizes, races, and the pathophysiology of IOP increases. Although elevated IOP is common in GO patients [8], the optic disc or visual field damage characteristics of glaucoma were not observed in this study.

Similar to the study of Sara et al., there were more male patients than female patients [16]. In our study, the range of IOP was wider for male patients and left eyes maybe because there were more male patients and more left eye cases.

This study showed that many eyes classified as phase 4 (36 eyes, 64.1%) and phase 6 (20 eyes, 34.5%) in the NOSPECS system exhibited elevated IOP, which is similar to other reports of proptosis and ocular movement disorders in GO accompanied by elevated IOP [8, 13]. However, the distribution of high IOP was wider, ranging from mild to sight-threatening, in the EUGOGO system. In addition, most eyes with high IOP in the mild and moderate-severe categories were classified as phase 4 in the NOSPECS system, which suggests that the use of both the EUGOGO and NOSPECS systems is beneficial for monitoring IOP in clinical practice. The IOP should also be monitored, even in mild cases of GO, according to the EUGOGO classification, especially when extraocular muscles are involved according to the NOSPECS classification.

In this study, elevated IOP was rarely observed in NOSPECS phase 3 eyes (only 1 eye), which is slightly different from the report by Behrouzi [8], who found that IOP increases, including open-angle glaucoma and suspected glaucoma, were not uncommon in phase 3 eyes, in addition to being present in phase 4 and phase 6 eyes.

In this study, increased IOP was observed not only in the active eyes but also in the inactive eyes. Behrouzi et al. [8] and Konuk et al. [17] also found that IOP may remain elevated despite disease remission. Additionally, chronic and persistent episcleral venous pressure may be elevated due to secondary changes in these structures [18] or due to a compromised aqueous drainage system [8]. The finding that IOP in the inactive eyes was similar to that in the active eyes in this study profoundly supports the above presumption.

Cockerham et al. [8, 13] showed that ophthalmometric values were >20 mm in 60–77% of GO patients. Other reports have shown that the extent of IOP elevation may be correlated with the degree of proptosis [19, 20]. This study also showed that IOP elevation tended to be associated with increased proptosis.

No glaucomatous optic changes or visual field defects were detected in this study. Forte et al. [21] did not find glaucomatous perimetric defects either; however, they found glaucomatous excavation of the optic disc and retinal nerve fiber layer thinning in some cases of GO with intraocular hypertension using OCT. In addition, Behrouzi et al. [8] reported visual field changes in some cases of compressive optic neuropathy with high IOP. All of the above data indicate that persistent IOP elevation may result in the progression of glaucomatous neuropathy. The absence of glaucomatous optic changes and visual field defects in this study may be due to the insufficient duration of elevated IOP for the manifestation of optic nerve damage.

Goldmann applanation tonometry (GAT) is the gold standard for measuring IOP. However, an NCT was used in this study. The NCT utilizes an air jet to achieve corneal flattening based on the same principle that GAT applies to measure IOP [22]. The main advantages of an NCT over GAT include that it is noninvasive and more convenient, thus facilitating patient cooperation, and no direct contact with the cornea occurs, which decreases its consequences [23, 24] and lessens any influence on tear film instability in GO patients [25]. In addition, many studies have reported no significant differences between IOP measurements obtained by GAT and an NCT [24, 26]. In another study, a Tono-Pen tonometer was also used to measure IOP in GO patients [27].

## 4. Conclusions

In conclusion, increased IOP was present in eyes with every degree of severity and disease activity of GO in the EUGOGO system, which showed a broader distribution than the NOSPECS system. Although no glaucomatous optic changes or visual field defects were detected in this study, routine IOP measurements and ophthalmic examinations are recommended to identify glaucomatous damage during follow-up examinations, regardless of the activity and severity of GO.

## Figures and Tables

**Table 1:** Demographic characteristics of elevated intraocular pressure of GO patients.

Characteristic	
Gender (male/female)	21/18
Eye (right/left)	28/30
Smoking (yes/no)	10/29
Age (mean ± SD, range, years)	49.8 ± 12.5 (25–75)
Male patients	49.5 ± 14.4 (25–75)
Female patients	50.2 ± 10.4 (27–67)
Duration (mean ± SD, range, months)	
Thyroid disease	15.9 ± 18.9 (1–96)
Graves' orbitopathy	7.5 ± 6.2 (1–36)
Diagnosis of primary thyroid disease (number, %)	
Hyperthyroidism	36 (92.3)
Thyroid cancer	1 (2.6)
Euthyroidism.	2 (5.1)
Treatment of thyroid disease (number, %)	
Antihyperthyroidism drugs	28 (71.8)
I^131^	7 (17.9)
Thyroidectomy	4 (10.3)
Current thyroid function (number, %)	
Normal	1 (2.6)
Hyperthyroidism	34 (87.2)
Hypothyroidism	3 (7.7)
Unrecorded	1 (2.6)

**Table 2:** Comparison of intraocular pressure (IOP) in severities of Graves' orbitopathy.

	Number of eyes	IOP (mmHg)
Mild	8	23.72 ± 3.33
Moderate-severe	29	25.57 ± 7.04
Sight-threatening	21	24.24 ± 2.55
P ^*∗*^	/	0.417
Phase 3	1	22
Phase 4	36	25.25 ± 6.49
Phase 5	1	22
Phase 6	20	24.35 ± 2.56
P^^^	/	0.463

^*∗*^The Kruskal–Wallis test; ^^^independent samples *t*' test, data of IOP in phases 3 and 5 deleted for statistics due to too little sample size in them.

**Table 3:** Comparison of elevated intraocular pressure (IOP) in different degrees of proptosis.

Degree of exophthalmos	Number of eyes (%)	IOP (mmHg)
Absent (normal–20 mm)	32 (55.2)	23.88 ± 2.65
Mild (21 mm–23 mm)	15 (25.9)	24.93 ± 5.05
Moderate (24 mm–27 mm)	5 (8.6)	25.60 ± 4.72
Severe (≥28 mm)	6 (10.3)	29.00 ± 12.85
P ^*∗*^	/	0.721

^*∗*^The Kruskal–Wallis test.

## Data Availability

The datasets used and/or analyzed during the current study are available from the corresponding author upon reasonable request.

## Abbreviations

GO: Graves' orbitopathy
IOP: Intraocular pressure
EUGOGO: European Group of Graves' Orbitopathy
CAS: Clinical activity score
NCT: Noncontact tonometer
GAT: Goldmann applanation tonometry.
